# Antiproliferative and proapoptotic activities of anthocyanin and anthocyanidin extracts from blueberry fruits on B16-F10 melanoma cells

**DOI:** 10.1080/16546628.2017.1325308

**Published:** 2017-06-19

**Authors:** Erlei Wang, Yanjun Liu, Caina Xu, Jingbo Liu

**Affiliations:** ^a^ College of Food Science and Engineering, Jilin University, Changchun, Jilin, PR China; ^b^ Key Laboratory of Polymer Ecomaterials, Changchun Institute of Applied Chemistry, Chinese Academy of Sciences, Changchun, Jilin, PR China

**Keywords:** Blueberry, anthocyanins, anthocyanidins, cell apoptosis, cell cycle

## Abstract

**Background**: Anthocyanins have been proven to affect multiple cancer-associated processes in different cancer cell lines. However, relatively few studies have investigated the effects of blueberry anthocyanins on metastatic melanoma. Thus, this study focuses on evaluating the chemopreventive potential of blueberry anthocyanins and their aglycones (anthocyanidins) in B16-F10 melanoma cells.

**Methods**: Blueberry anthocyanin and anthocyanidin extracts were prepared mainly by combined chromatography techniques. Their antiproliferative and proapoptotic effects on B16-F10 cells were evaluated by MTT assay, calcein acetoxymethyl ester/propidium iodide (calcein-AM/PI) staining, and flow cytometry of the cell cycle and apoptosis.

**Results**: The MTT and calcein-AM/PI staining results showed that both anthocyanin (purity of 62.5%) and anthocyanidin (75.1%) extracts could significantly inhibit the viability and proliferation of B16-F10 cells in a dose-dependent manner, while anthocyanidin extracts exhibited significantly higher (*p* < 0.05) cytotoxicity than anthocyanin extracts. Furthermore, anthocyanin and anthocyanidin extracts blocked cell cycle procession at the G0/G1 phase below 400 and 200 μg/mL, and induced early apoptosis below 400 and 300 μg/mL, respectively.

**Conclusions**: These data suggest that both anthocyanin and anthocyanidin extracts inhibit the proliferation and trigger the apoptosis of B16-F10 cells, and anthocyanidin extracts may be a more promising candidate in preventing metastatic melanoma than anthocyanin extracts.

## Introduction

Metastasis is a complex multistep process by which malignant tumors can spread to distal organs [1]. Tumor metastasis is regarded as the most common cause of mortality in cancer patients. Various anticancer drugs have been widely used for cancer therapy, such as doxorubicin, lovastatin, exisulind, and docetaxel [2]. However, nearly all anticancer drugs have potential or life-threatening side effects. Therefore, it is necessary to find more effective anticancer agents with minimal toxic effects.

Epidemiological as well as *in vivo* clinical studies have shown that the consumption of diets rich in fruits and vegetables is beneficial to the prevention of certain types of human cancer [3]. It is believed that phytochemicals such as polyphenols in fruits or vegetables can induce cell apoptosis or cell cycle arrest in tumor-derived cells, while having a relatively low toxicity [4]. Anthocyans, one kind of the dietary polyphenol, are widely distributed in fruits, beans, cereals, and vegetables, and suggested to be associated with reduced risk of human breast cancer [5], human colon cancer [6], and human ovarian cancer [7]. Anthocyans are composed of two types, anthocyanins and anthocyanidins. Anthocyanins are regarded as the largest group of water-soluble pigments in the plant kingdom, and are glycosylated or acylglycosylated forms of polyhydroxy or polymethoxyl derivatives of 2-phenylbenzopyrylium [8]. The de-glycosylated or de-acyl-glycosylated forms of anthocyanins are called anthocyanin aglycones or anthocyanidins [9]. The most common anthocyanidins found in plants include delphinidin (De), cyanidin (Cy), petunidin (Pt), peonidin (Pn), pelargonidin (Pg), and malvidin (Mv) [10]. A previous report demonstrated that anthocyanidins caused stronger growth inhibition in human hepatoma cell lines than anthocyanins; furthermore, anthocyanidins exhibited more effective inhibitory effects against HepG2 cells than Hep3B cells [11]. Another report showed that not only anthocyanidins but also anthocyanins inhibited cancer cell growth and induced apoptosis in cancer cells [12]. Some other studies found that anthocyanins and their aglycones selectively inhibited the growth of cancers, but exerted little or no effect on the growth of normal cells [13,14]. The structural differences between anthocyanins and anthocyanidins may lead to significant differences in their anticancer efficiency, antioxidant activities, bioavailability, and other biological effects [15]. In addition, it should be noted that the anticancer effects of anthocyans may vary with different cancer cell lines.

Among fruits and vegetables, blueberries have been considered to be one of the fruits with the highest anthocyanin content. The high anthocyanin content of blueberries may contribute to the health benefits against chronic diseases including cancers. Yi and co-workers reported that blueberry anthocyanins induced apoptosis in HT-29 and Caco-2 cells, and resulted in a two- to seven-fold increase in DNA fragmentation [16]. Faria et al. demonstrated that blueberry anthocyanin extracts significantly reduced the proliferation of two breast cancer cell lines (MDA-MB-231 and MCF7) and exhibited obvious anti-invasive potential in both cell lines [17].

It is well known that B16-F10 melanoma cells are a highly invasive metastatic cell line, and finding a cure for metastatic melanoma remains a challenge for experimental and clinical oncology. Recently, the anthocyanin-rich fraction of blueberries was found to inhibit proliferation, stimulate apoptosis, and increase lactate dehydrogenase leakage activity in B16-F10 melanoma murine cells [18]. In another study, mulberry anthocyanin extract was proven to prevent atherosclerosis and inhibit melanoma metastasis [19]. Although a few studies have demonstrated the antitumor activities of blueberry anthocyanins, most of the research focused on glycosylated anthocyanins, and information on anthocyanin aglycones is still limited. *Vaccinium uliginosum* L. is a wild low-bush blueberry species native to China, and has various biological activities including notable antioxidant activity [20]. Our previous research indicated that the fruits were rich in anthocyanins, and malvidin-3-*O*-glucoside, petunidin-3-*O*-glucoside, and delphinidin-3-*O*-glucoside were identified as the three major anthocyanin monomers [21].

**Scheme 1.**
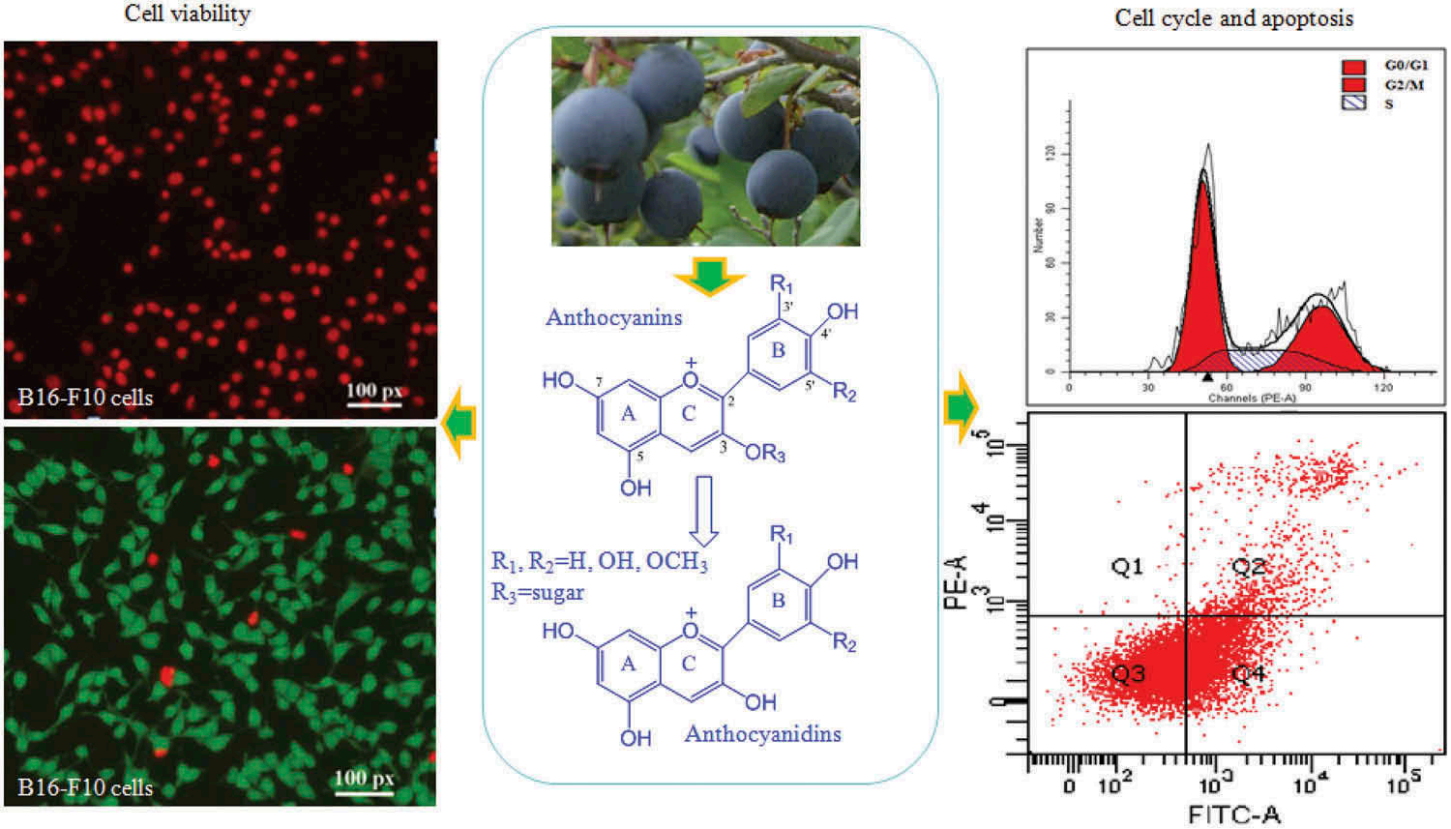
Experimental design for evaluating the antiproliferative and proapoptotic effects of blueberry anthocyanins and anthocyanidins on B16-F10 cells.

The objective of this study was to separate anthocyanin and anthocyanidin extracts from blueberry fruits, and examine the inhibitory effects of both extracts against B16-F10 melanoma murine cells. A further aim was to assess the cell viability, cell cycle, and apoptosis (Scheme 1).

## Materials and methods

### Reagents

Chromatographic grade methanol (MeOH) was supplied by Merck (Darmstadt, Germany). Ethanol, ethyl acetate (EtOAc), and hydrochloric acid (HCl) were purchased from Beijing Chemistry Factory (Beijing, China). Doxorubicin hydrochloride was purchased from Beijing Huafeng United Technology Co. (Beijing, China). Standards of cyanidin-3-*O*-glucoside chloride [molecular weight (MW) 484.84, purity ≥ 98%] and cyanidin chloride (MW 322.7, purity ≥ 97%) were purchased from Sigma-Aldrich Chemical Co. (St Louis, MO, USA).

### Preparation of blueberry anthocyanin extracts

Low-bush wild blueberries (*V*. *uliginosum* L.) were collected in August 2015 from naturally occurring woodlands situated in Changbai Mountain zones (Jilin province, China). First, 1 kg of blueberry fruit samples was extracted with 2 L of 70% aqueous ethanol containing 0.1% HCl for 24 h at room temperature in the dark. The extract was centrifuged at 3000 rpm for 5 min, and the residue was re-extracted twice with 2 L of aqueous ethanol for 24 h. The organic solvent of the pooled supernatants was evaporated below 50°C using a rotary evaporator (RE-52A; Yarong, Shanghai, China). The concentrated liquid was partitioned against EtOAc three times to remove pectins and non-anthocyanin phenolics. The organic phase was removed and the water phase was applied to an Amberlite XAD-7HP column (2.6 cm × 50 cm; particle size: 20–60 mesh; wet; Sigma-Aldrich). The column was initially washed with 1.5 L of deionized water (0.01% HCl) at a flow rate of 1 mL/min to remove the majority of free sugars, proteins, and organic acids, and then eluted with 0.6 L of 30% aqueous ethanol containing 0.01% HCl at a flow rate of 1.5 mL/min, followed by 0.6 L of 80% ethanol/water at a flow rate of 1.5 mL/min to remove the absorbed impurities on the column. The eluate of 40% aqueous ethanol was concentrated by rotary evaporation below 50°C to remove ethanol. The concentrated extract was applied for activating C18 Sep-Pak cartridges (Waters, Milford, MA, USA) to adsorb anthocyanins. After washing of the cartridge with 0.01% HCl in aqueous solution water, the anthocyanins fraction was collected by elution with 3 mL of 50% ethanol in deionized water (containing 0.01% HCl), concentrated under vacuum below 50°C, and freeze-dried in a vacuum freeze-dryer (FD-1A-50; Boyikang, Beijing, China). Afterwards, the dried extract was stored at −80°C for further studies.

### Preparation of blueberry anthocyanidin extracts

The dried anthocyanin extracts (~200 mg) obtained by the Amberlite XAD-7HP column were hydrolyzed in a screw-cap test-tube with 100 mL of 2.5 mol/L HCl, flushed with nitrogen and capped as previously described [22]. The anthocyanins were hydrolyzed for 1 h at 95°C and then immediately cooled in an ice bath. The hydrolysate was concentrated using a rotary evaporator below 50°C. The concentrate was adsorbed on to the C18 Sep-Pak cartridge (Waters) previously activated with 2.0 mL methanol and 2.0 mL of 0.01% aqueous HCl. The cartridge was rinsed with 2.0 mL of 0.01% aqueous HCl to remove sugars, acids, and other water-soluble compounds. Anthocyanidins were recovered with 60% aqueous ethanol containing 0.01% HCl (v/v). The ethanol was evaporated using a rotary evaporator below 50°C and freeze-dried in a vacuum freeze-dryer. The dried anthocyanidin extract was stored at −80°C for further characterization and cell-based studies.

### High-performance liquid chromatography–diode array detector (HPLC-DAD) analysis of anthocyanins and anthocyanidins

The profiles of anthocyanins and anthocyanidins were analyzed on a Shimadzu HPLC system (LC-30AD; Shimadzu) with a DAD (SPD-M20A; Shimadzu) at 520 nm. Two milligrams of the samples was dissolved in 3% formic acid and filtered through a 0.22 μm polytetrafluoroethylene (PTFE) filter membrane, and HPLC was performed on a Waters Symmetry Shield C18 column (4.6 mm × 150 mm, 5 μm). The column temperature was set at 25°C with the elution solvent A (100% methanol) and solvent B (3% formic acid in water, v/v). The gradient was: 0–5 min, 85% B; 6–10 min, 85–80% B; 11–25 min, 80–75% B; 26–32 min, 75% B; 33–39 min, 75–30% B; 40–45 min, 30% B; 46–55 min, 30–85% B; 56–60 min, 85% B. The flow rate was 0.5 mL/min and the injection volume was 10 μL.

For the quantitative analysis, the HPLC-DAD system was applied to determine the total contents of anthocyanins and anthocyanidins in purified blueberry samples using a calibration curve by analyzing different concentrations (1–100 μg/mL) of commercial standards of anthocyanins (cyanidin-3-*O*-glucoside) and anthocyanidins (cyanidin). Since most of the monomeric anthocyanins or anthocyanidins have similar spectroscopic properties and a comparable molecular mass, the contents of anthocyanins and anthocyanidins can be expressed in terms of the standards [23]. The peak areas of all the components in the HPLC chromatogram at 520 nm were summed and measured against the pure standards.

### Cell culture

B16-F10 metastatic murine melanoma cell lines and mouse fibroblast L929 cells were obtained from the American Type Culture Collection (Rockville, MD, USA). B16-F10 cells were cultured in Dulbecco’s modified Eagle’s medium (DMEM) containing 10% heat-inactivated fetal bovine serum, 1 g/L glucose, 1 mmol/L glutamine, 100 U/mL penicillin, and 100 μg/mL streptomycin. L929 cells were used for cytotoxicity analysis of anthocyanins or anthocyanidins. L929 cells were cultured in ATCC medium (McCoy’s 5a medium with 1.5 mM L-glutamine, 90%; fetal bovine serum, 10%). Incubation was carried out at 37°C in a humidified atmosphere of 95% air and 5% carbon dioxide. The medium was changed every day.

### Analysis of cell viability

Cell viability was assessed by the 3-[4,5-dimethylthiazol-2-yl]-2,5-diphenyl-tetrazolium bromide (MTT) colorimetric assay. B16-F10 and L929 cells were seeded at a density of 8 × 10^3^ cells/well on 96-well plates. After 24 h incubation, anthocyanin and anthocyanidin extracts at different concentrations (12.5, 25, 50, 100, 200, 400, 600, and 800 μg/mL) were dissolved in phosphate-buffered saline (PBS) containing 0.5% dimethylsulfoxide (DMSO) and added to cells in each well and then incubated for 24, 48, and 72 h, respectively. Different concentrations of doxorubicin (0.04, 0.08, 0.16, 0.32, 0.64, 1.25, 2.5, and 5.0 μg/mL) were used as the positive control. After incubation for 48 h, 20 μL of MTT (5 mg/mL in PBS) was added to each well and incubated for 4 h at 37°C. After the supernatant was removed, 160 μL of DMSO was added to each well to solubilize the produced fromazan crystals, and measurements were taken at 490 nm using a Bio-Rad 680 Microplate reader. Cell viability was expressed as a percentage of the control culture value, which was considered as 100% viable.

### Live/dead assay by calcein acetoxymethyl ester/propidium iodide (calcein-AM/PI) staining

The effects of anthocyanins and anthocyanidins on the viability or cytotoxicity of B16-F10 cells were evaluated using the Calcein-AM/PI Double Stain Kit (Molecular Probes, Eugene, OR, USA). This kit stains both living and dead cells simultaneously using two fluorescent dyes. Living cells produce green fluorescence because of the conversion of calcein acetoxymethyl ester (calcein-AM) to calcein, while dead cells produce red fluorescence owing to the presence of propidium iodide (PI). In brief, B16-F10 cells were seeded at 8 × 10^3^ cells/well into 96-well plates, as above. After leaving overnight, anthocyanin and anthocyanidin extracts at different concentrations (25, 50, 100, 200, 400, and 800 μg/mL) were added to the medium in each well. After incubation for 24 h, the medium in each well was removed and the cells were labeled with the Calcein-AM/PI Kit. Finally, the living cells (green cytoplasmic fluorescence) and dead cells (red nucleus fluorescence) were observed by an inverted fluorescence microscope.

### Cell cycle by flow cytometric analysis

The B16-F10 cells were seeded into six-well plates at a density of 1 × 10^5^ cells/mL. After incubation for 24 h, the cells were treated with various concentrations of anthocyanin and anthocyanidin extracts (0, 50, 100, 200, 300, 400, and 800 μg/mL). Untreated cells were also included in this experiment for comparison. The cell cycle distribution was measured by flow cytometry based on the incorporation of labeled precursors in DNA contents. DNA content is changed with the process of the cell cycle, which can be used to calculate the percentage of G_0_/G_1_, S, and G_2_/M. A Cell Cycle Detection Kit (KeyGen BioTech Co., Nanjing, China) was used to distinguish between cells in different stages of the cell cycle. Flow cytometric analysis was conducted using the FACSCalibur (BD Biosciences; San Jose, CA, USA) and data were analyzed using FACSDiva Software (version 6.1.3; BD Biosciences).

### Cell apoptosis by flow cytometric analysis

The changes in cell apoptosis were detected using an Annexin V-FITC/PI Apoptosis Detection Kit (KeyGen BioTech Co., Nanjing, China) and subsequent flow cytometry. In brief, the B16-F10 cells were seeded into six-well plates at a density of 1 × 10^5^ cells/mL. After incubation for 24 h, the cells were treated with different concentrations of anthocyanin and anthocyanidin extracts (0, 50, 100, 200, 300, 400, and 800 μg/mL). After incubation for 24 h, the cells were trypsinized and stained with Annexin V-FITC and PI in the dark. Then, the cells were collected from each well, followed by flow cytometry for the fluorescence intensity, with the FL-1H channel detecting fluorescein isothiocyanate (FITC) at the wavelengths of 488 and 530 nm. The untreated B16-F10 cells were used as the negative control.

### Data analysis

Data are presented as arithmetic mean ± standard deviation (SD). Statistical analyses between various groups were performed by one-way analysis of variance (ANOVA). Statistical differences between samples were evaluated by Tukey’s multiple comparison test. Significance was considered at *p *< 0.05 in all analyses.

## Results

### Identification of anthocyanins and anthocyanidins in purified blueberry anthocyan extracts

**Figure 1.**
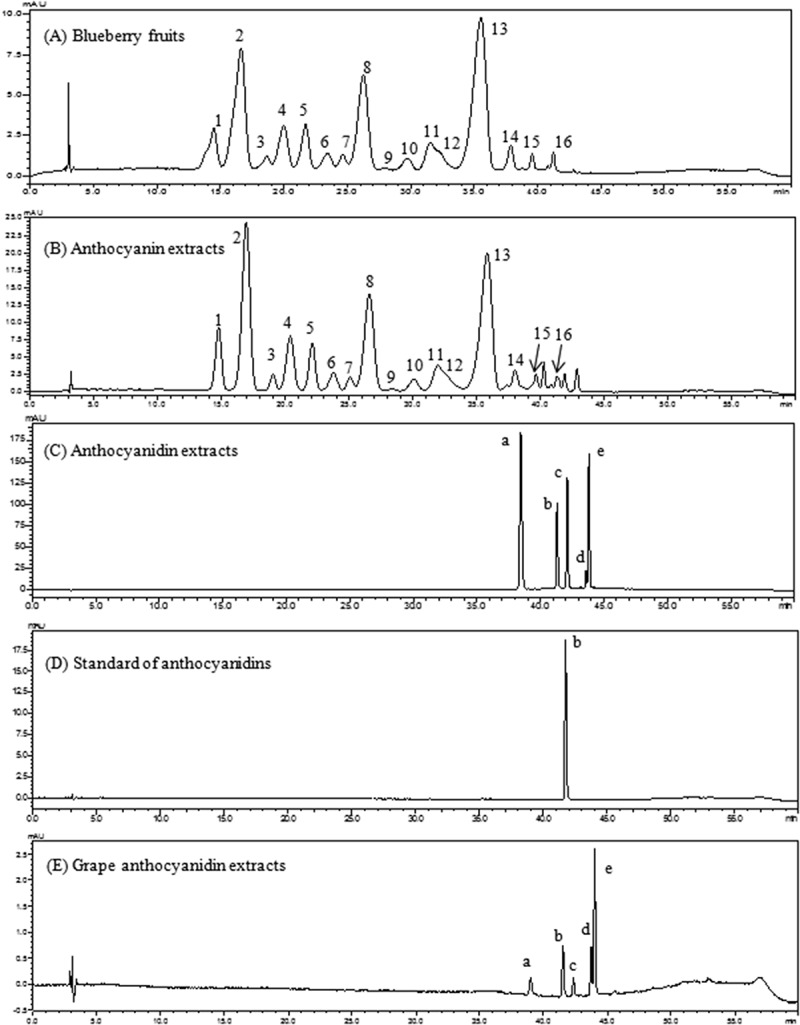
High-performance liquid chromatography chromatograms of anthocyanins and anthocyanidins from blueberry fruits and purified extracts detected at 520 nm. 1, Delphinidin-3-*O*-galactoside; 2, delphinidin-3-*O*-glucoside; 3, cyanidin-3-*O*-galactoside; 4, delphinidin-3-*O*-arabinoside; 5, cyanidin-3-*O*-glucoside; 6, petunidin-3-*O*-galactoside; 7, cyanidin-3-*O*-arabinoside; 8, petunidin-3-*O*-glucoside; 9, peonidin-3-*O*-galactoside; 10, petunidin-3-*O*-arabinoside; 11, peonidin-3-*O*-glucoside; 12, malvidin-3-*O*-galactoside; 13, malvidin-3-*O*-glucoside; 14, malvidin-3-*O*-arabinoside; 15, petunidin-3-*O*-xyloside; 16, malvidin-3-*O*-xyloside; a, delphinidin; b, cyanidin; c, petunidin; d, peonidin; e, malvidin.

The chemical structures of anthocyanins and anthocyanidins in the two anthocyan samples were confirmed through the combined interpretation of ultraviolet–visible spectra, predicted molecular polarities, congruent retention time with the available standards, HPLC-DAD–electrospray ionization–tandem mass spectrometry data, and the identities of anthocyans in other blueberry species comparable to those in earlier studies [24]. The details of the identification procedure have been reported in our previous publication [21]. Consequently, a total of 16 major anthocyanin peaks was identified in blueberry fruits (Figure 1(A)). Following the separation procedures of extraction, partition, column chromatography, and solid-phase extraction, the anthocyanin extracts were obtained, and the HPLC profiles in Figure 1(B) indicated that nearly all the 16 monomeric anthocyanins were obtained, despite a slight difference in the ratios of peak areas of each anthocyanin compared with the anthocyanin profiles in blueberry fruits (Figure 1(A)). In the purified anthocyanin extracts, the average content of five anthocyanins followed the order of De > Mv > Pt > Cy > Pn. The De-based derivatives (De, Pt, and Mv) were more abundant than the Cy-based derivatives (Cy and Pn). For instance, the three prominent monomeric anthocyanins (peaks 2, 8, and 13 in Figure 1(B)) represented nearly 63.5% of the total peak area, and the most abundant monomeric anthocyanin was determined to be delphinidin-3-*O*-glucoside (peak 2, 25.5%). Using the improved elution parameters in the column chromatography and the newly introduced solid-phase extraction, the anthocyanin extracts were obtained with a purity of 62.5% (w/w), compared with 32.0% (w/w) in the crude extracts in our previous report [21]. The highly purified anthocyanin extracts could find more extensive application as a bioactive ingredient compared with the crude extract in our previous studies or the similar extracts (17.6%, w/w) from blueberries in a 2014 publication [25].

Anthocyanidin extracts were obtained by acid hydrolysis of blueberry anthocyanin extracts and purified by solid-phase extraction. The HPLC chromatograms are presented in Figure 1(C). Four major peaks and one small peak were observed in the anthocyanidin extracts. To confirm the profiles of the five peaks in the obtained extract, an authenticated standard (Cy) and a local Kyoho grape variety were used as references. The anthocyanidins from grape skin extracts have been extensively studied and characterized as five basic aglycones of De, Cy, Pt, Pn, and Mv [15]. The grape sample was extracted and hydrolyzed in the same way as the blueberry samples. Figure 1(D) shows the retention time of Cy standard (41.6 min), which matched the retention time of Cy in Figure 1(C). In addition, the identities of five basic aglycones in Figure 1(C) can be confirmed (De, Cy, Pt, Pn, and Mv) using grape (Figure 1(E)) as a reference [26]. The three aglycones (De, Pt, and Mv) represented 81% of the total peak area percentage in anthocyanidin extracts, whereas Cy and Pn were the two minor aglycones. After solid-phase extraction, concentration, and freeze-drying, the anthocyanidin contents in the purified extract reached 75.1% (w/w) compared to 62.5% (w/w) in the anthocyanin extracts. The blueberry anthocyanins were further applied to the evaluation of bioactivity in cells.

### Cytotoxicity of anthocyanin and anthocyanidin extracts

**Figure 2.**
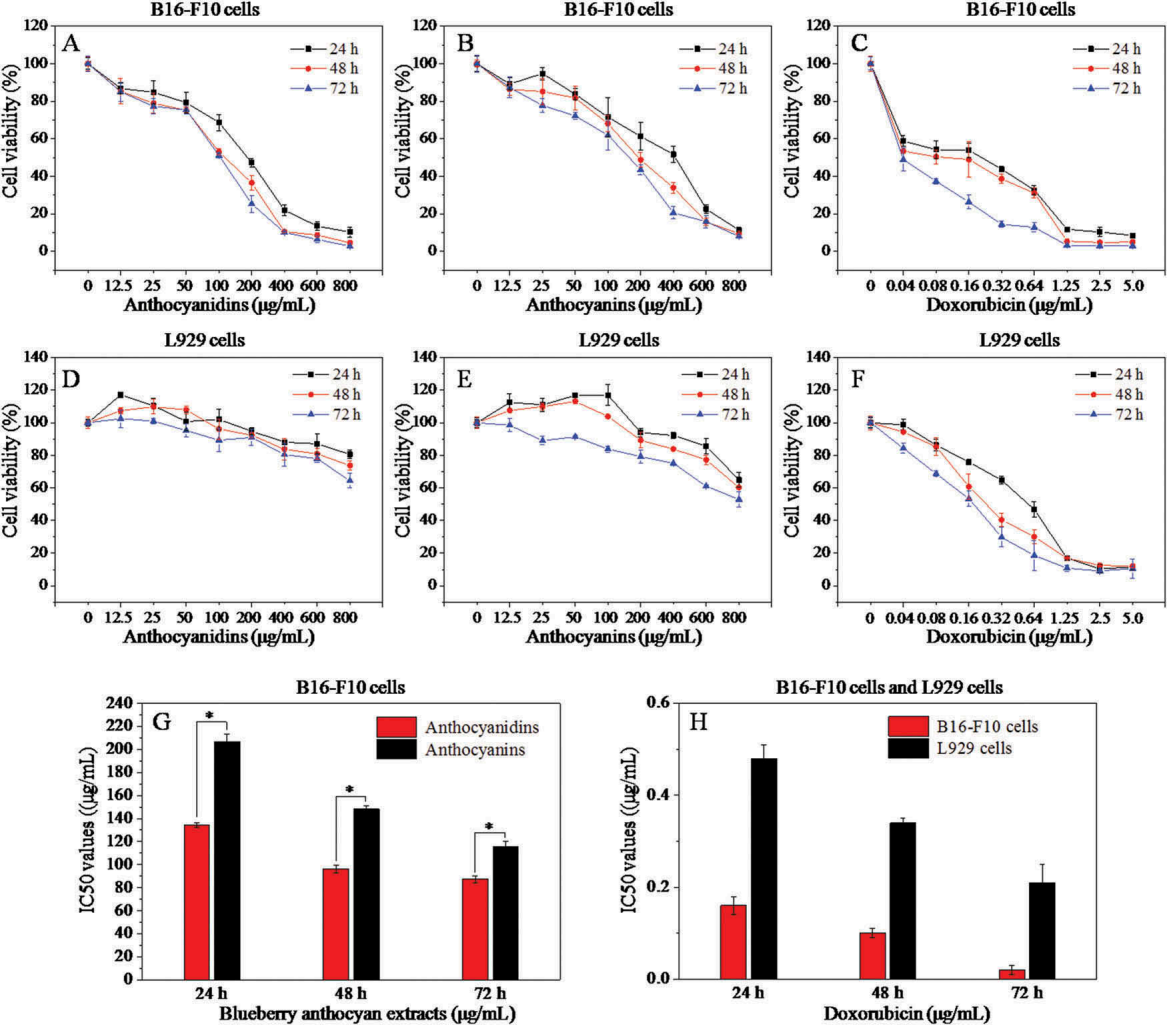
Effects of different concentrations of anthocyanidins, anthocyanins, and doxorubicin on the cell viability of B16-F10 and L929 cells at 24 h, 48 h, and 72 h based on the MTT assay (A–F), and the corresponding median inhibitory concentration (IC_50_) values (G, H). Data are presented as mean ± SD (*n *= 6). *p < 0.05 compared with the anthocyanidin-treated group.

The influence of anthocyanin and anthocyanidin extracts on the growth of B16-F10 and L929 cells at 24, 48, and 72 h was investigated using the MTT assay (Figure 2). Both anthocyanidins and anthocyanins inhibited the growth of B16-F10 cells within a broad concentration range (Figure 2(A,B)). In general, lower concentrations (12.5 and 25 μg/mL) of both samples had a slight inhibitory effect on the proliferation of B16-F10 cells compared with the blank control (0 μg/mL), although the cell viability was much higher than 50%. However, when the concentration of anthocyanins ranged from 50 to 800 μg/mL and anthocyanidins from 50 to 400 μg/mL, the cell viability decreased sharply in a dose-dependent manner. Blueberry anthocyanidins showed a significantly higher (*p* < 0.05) toxicity than that of anthocyanins after 24 h treatment on B16-F10 cells, with median inhibitory concentration (IC_50_) values of 134.1 and 206.7 μg/mL, respectively (Figure 2(G)). Subsequently, anthocyanidins were more cytotoxic than anthocyanins at 48 and 72 h, with IC_50_ values of 96.20 and 87.33, and 148.4 and 115.6 μg/mL, respectively. Derived from the normal tissue of the mouse, L929 cells are commonly used as a reference cell line for testing the cytotoxicity of polymers or natural plants. Blueberry anthocyanidins and anthocyanins at a concentration of 12.5–800 μg/mL did not have a marked effect on the cell viability of L929 cells (Figure 2(D,E)) after incubation for 24, 48, and 72 h, suggesting that the two extracts showed low or no cytotoxicity on normal cells but a highly efficient inhibitory effect on B16-F10 metastatic murine melanoma cells under *in vitro* conditions. Doxorubicin, a commercial drug, was used as a positive control in this study (Figure 2(C)). Doxorubicin appeared to be the most toxic drug on B16-F10 cells, with IC_50_ values of 0.16, 0.10, and 0.02 μg/mL at 24, 48, and 72 h (Figure 2(H)), respectively. Similarly, doxorubicin exhibited stronger cytotoxicity against L929 cells than two blueberry samples (Figure 2(F)), with IC_50_ values of 0.48, 0.34, and 0.21 μg/mL at 24, 48, and 72 h (Figure 2(F)), respectively. These results reveal that both anthocyanin and anthocyanidin extracts exhibited strong inhibitory effects against B16-F10 cells in a dose-dependent manner, and low or no cytotoxicity to L929 cells; furthermore, anthocyanidin extracts exhibited stronger cytotoxicity than anthocyanin extracts against B16-F10 cells.

### Calcein-AM/PI staining

**Figure 3.**
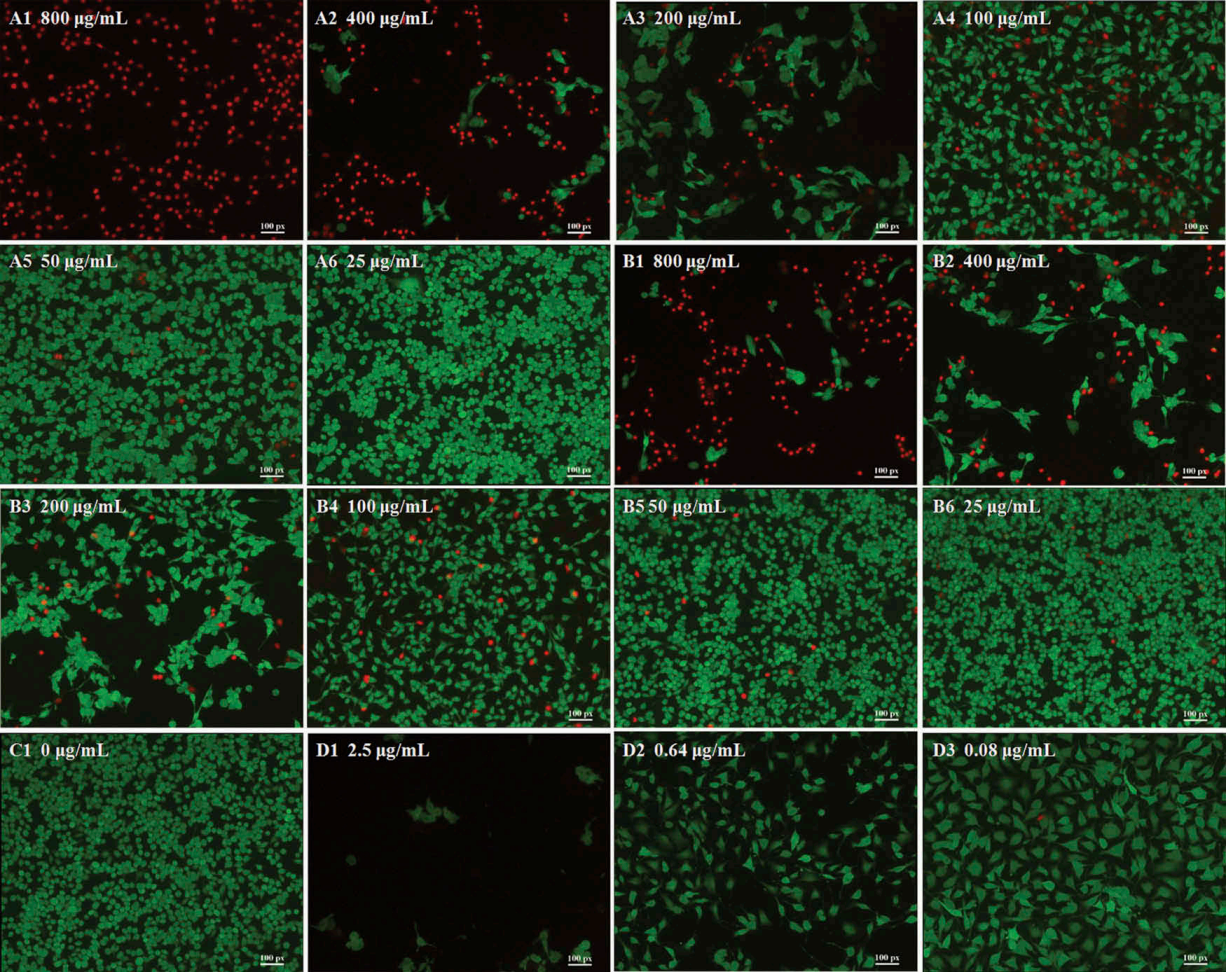
Morphology of B16–F10 cells by calcein acetoxymethyl ester/propidium iodide fluorescein staining. Cells were treated with anthocyanidins (A1–A6) and anthocyanins (B1–B6) at various concentrations (25–800 μg/mL) for 48 h. Non-treated B16-F10 cells served as the negative control (C1), while doxorubicin was the positive control (D1–D3). The fluorescence images were obtained by inverted fluorescence microscopy.

Cell viability and morphology can be measured using the fluorescent probes calcein-AM and PI, which can differentiate between living and dead cells [27]. In living B16-F10 cells, intracellular esterase can convert calcein-AM to calcein, which stays in living cells and emits intense green fluorescence. PI is cell membrane impermeable; it can only penetrate cells with impaired plasma membrane integrity, then bind with DNA and emit red fluorescence. The viability and morphology of B16-F10 cells were observed by fluorescence microscopy (Figure 3). Optical photography showed that the anthocyanidin and anthocyanin extracts obviously reduced the number and density of living cells at doses ranging from 25 to 800 μg/mL. Incubation of B16-F10 cells with higher doses of anthocyanidins (800 and 400 μg/mL in Figure 3A1 and A2) resulted in a drastic reduction in living cells stained with calcein (green) and an increase in the dead cells stained with PI (red) compared with the low-concentration group (50 and 25 μg/mL in Figure 3A5 and A6) and the untreated group (Figure 3C1). A similar phenomenon was also observed for treatments with anthocyanins (Figure 3B1–B6). As the red color reflects the cell membrane integrity, we speculated that the two blueberry anthocyan samples disrupted the cell membrane integrity. The trend in changed cell viability at different concentrations of anthocyan groups (Figure 3(A,B)) was generally similar with the doxorubicin group (Figure 3D1–D3); however, the living cells in the anthocyan groups had a spindle shape or an oval shape, in contrast to the unique polygon shape in the doxorubicin group, indicating that different inhibitors for B16-F10 cells could result in different morphological changes. It has been reported that apoptosis and necrosis are two patterns of cell death in multicellular organisms [28], and the cell death is accompanied by changes in cell morphology, such as cell shrinkage, rounding, and reticulum expansion [29,30]. Thus, we deduced that anthocyanins and anthocyanidins induced cell apoptosis or necrosis. Furthermore, the fluorescence microscopy results indicated that anthocyanidins had higher inhibitory effects on B16-F10 cells than anthocyanins, which is generally consistent with the MTT results.

### Cell cycle analysis by flow cytometry

**Figure 4.**
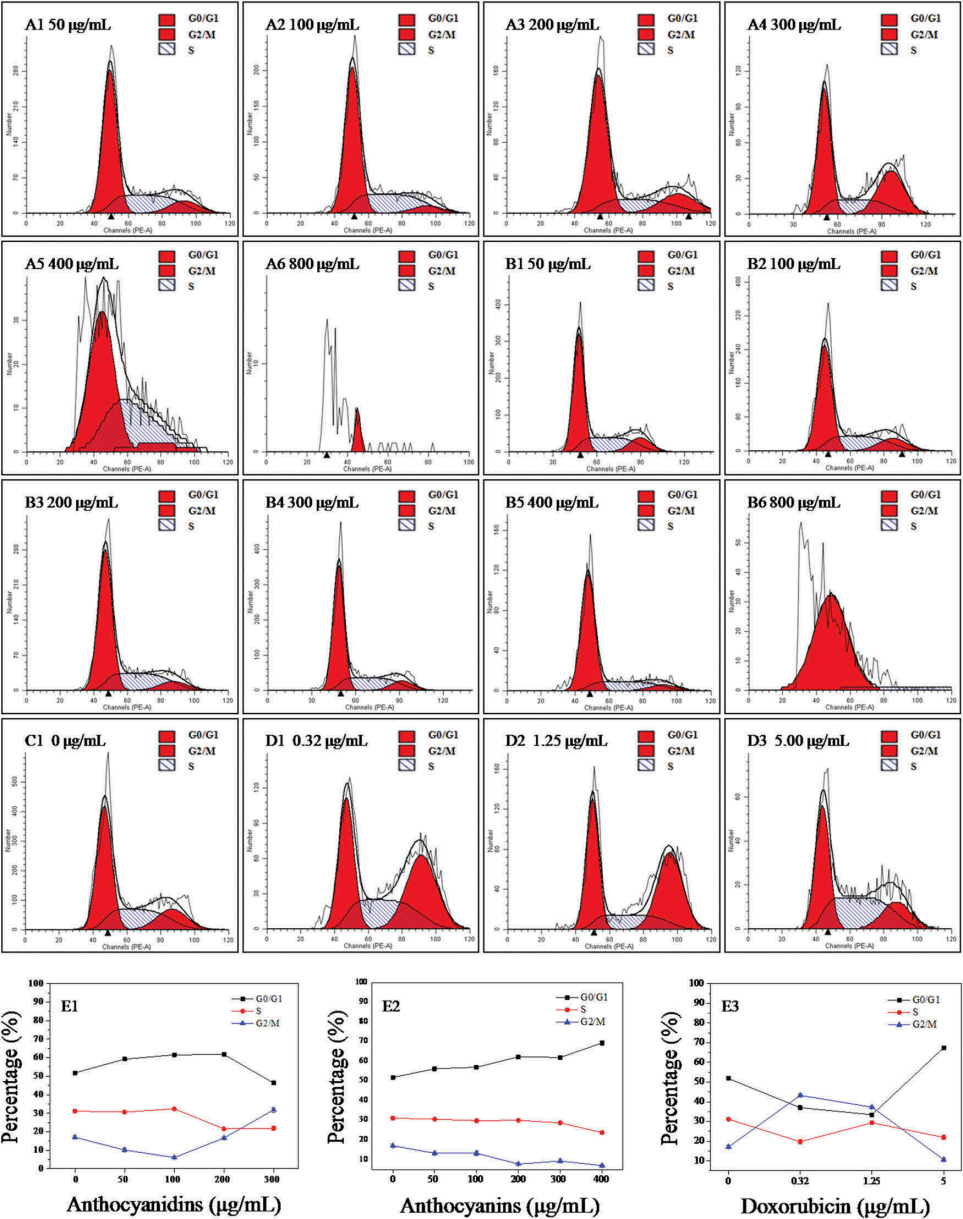
Flow cytometric analysis of the cell cycle in B16–F10 cells treated with blueberry anthocyanidins (A1–A6, E1) and anthocyanins (B1–B6, E2) at various concentrations (50–800 μg/mL) after 48 h. Non-treated B16–F10 cells served as the negative control (C1), while doxorubicin was the positive control (D1–D3, E3).

To analyze the cellular mechanism of growth inhibition of anthocyanins or anthocyanidins on B16-F10 cells, the cell cycle distribution was evaluated by flow cytometry. The B16-F10 cells were exposed to varying concentrations (0–800 μg/mL) of the two compounds for 48 h. As can be seen in Figure 4(A–E), the percentage distribution of the cells treated with 50 μg/mL anthocyanidins (Figure 4A1 and E1) was 59.2%, 30.5%, and 10.1% at the cell cycle G0/G1, S, and G2/M phases, respectively, compared to 51.8%, 31.1%, and 17.1% in the untreated cells (Figure 4C1 and E1). A continuously increasing percentage of cells in G0/G1 phase was also observed for treatments with higher concentrations (100 and 200 μg/mL) of anthocyanidins (Figure 4A2, A3 and E1). When the concentration of anthocyanidins exceeded 200 μg/mL, significant numbers of apoptotic or necrotic cells were observed (Figure 4A5 and A6), which could not actually reflect the cell cycle distribution. The present results indicate that blueberry anthocyanidins at a concentration of 50–200 μg/mL could cause an arrest of B16-F10 cells at the G0/G1 phase of the cell cycle, while at higher concentrations, a large number of cells was present in the G2/M phase. Compared to the anthocyanidins, blueberry anthocyanins at a concentration of 50–400 μg/mL could significantly delay the cell cycle by arresting cells in the G0/G1 phase (Figure 4B1–B5 and E2). Doxorubicin has a non-specific, broad-spectrum antitumor mechanism on the cell cycle, and can exert its activity independently of the specific phase of the cell cycle (Figure 4D1–D4 and E3). Together, these results suggest that blueberry anthocyanidins at a concentration of 50–200 μg/mL and anthocyanins at 50–400 μg/mL could arrest the B16-F10 cells at the G0/G1 phase, thus resulting in cell apoptosis.

As cyclin D1 is a key regulator of the G1 phase of the cell cycle [31], the levels of cyclin D1 were evaluated by immunofluorescence staining to examine the protein expressions of anthocyan-induced cell cycle block on the B16-F10 cells. The staining methods and relevant images are summarized in supplementary information (Figure S1), and green fluorescence intensity reflects cyclin D1 content in B16-F10 cells. The content of cyclin D1 decreased significantly (*p* < 0.05) in both anthocyanidin- and anthocyanin-treated groups compared with the control group (Figure S2); in turn, the anthocyan samples down-regulated cyclin D1 expression in the B16-F10 cells. Thus, the G0/G1 phase arrest in blueberry anthocyan-treated cells may involve the down-regulation of cyclin D1 expression.

### Cell apoptosis analysis by flow cytometry

**Figure 5.**
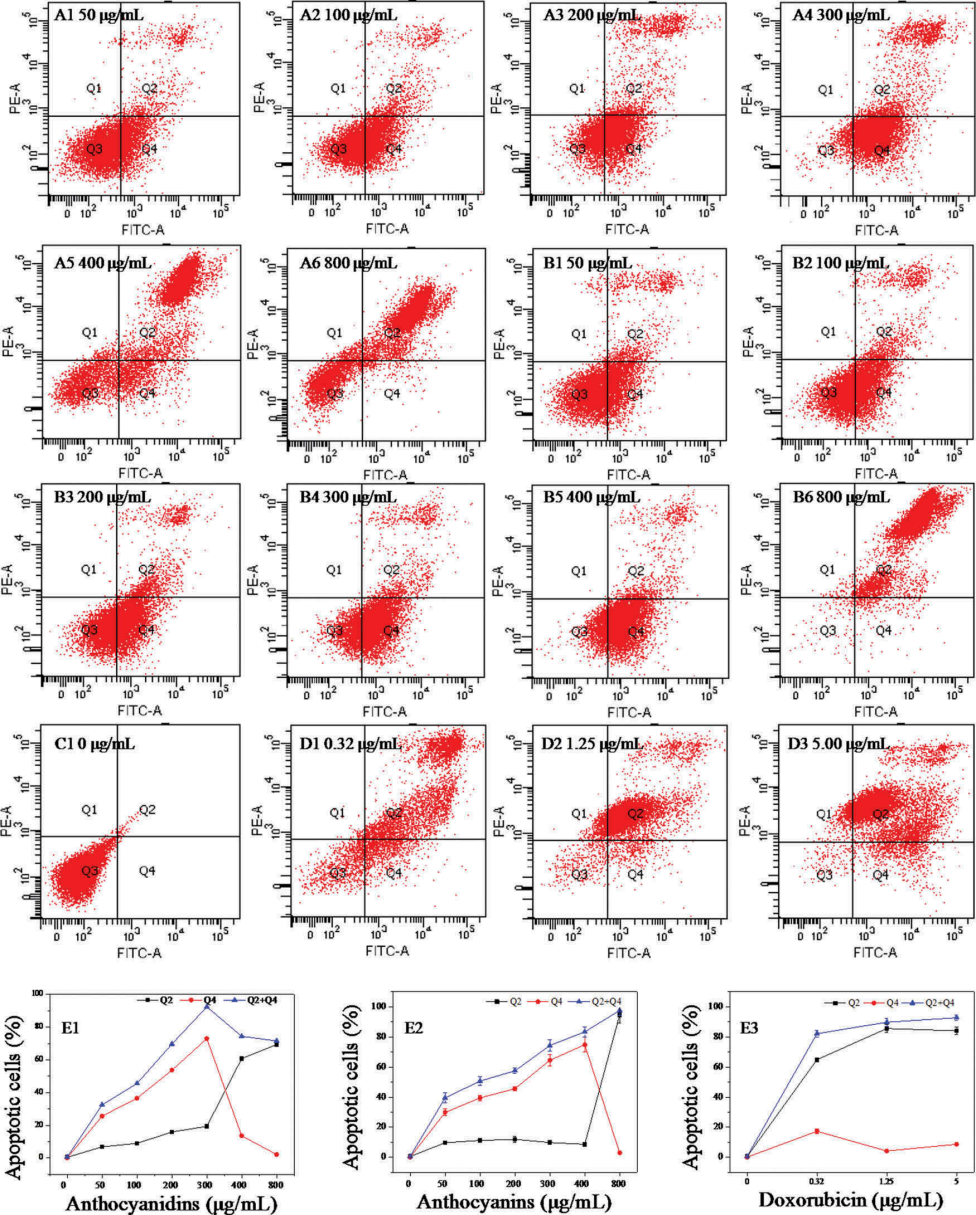
Annexin V–fluorescein isothiocyanate/propidium iodide flow cytometric analysis of cell apoptosis in B16–F10 cells in the presence of blueberry anthocyanidins (A1–A6, E1) and anthocyanins (B1–B6, E2) at various concentrations (50–800 μg/mL) after 48 h. Non-treated B16–F10 cells served as the negative control (C1), while doxorubicin was the positive control (D1–D3, E3). Q1, necrotic cells; Q2, cells in the stage of late apoptosis; Q3, viable cells; Q4, early apoptotic cells.

The B16-F10 cells were incubated with 0–800 μg/mL of blueberry anthocyanidins and of anthocyanins for 48 h. The cells were double stained with Annexin V-FITC/PI and subjected to flow cytometry. Figure 5(A–D) shows the apoptotic states of B16-F10 cells induced by anthocyanidins (Figure 5A1–A6) and anthocyanins (Figure 5B1–B6), compared with the negative control (Figure 5C1) and positive control (Figure 5D1–D3). Figure 5E1–E3 shows the proportions of B16–F10 cells in early apoptosis (Q4 quadrant in Figure 5A1–D3), late apoptosis (Q2 in Figure 5A1–D3), and total apoptotic cells (the sum of early and late apoptotic cells).

From Figure 5, the results show that B16-F10 cells exposed to 0, 50, 100, 200, and 300 μg/mL of anthocyanidins for 48 h induced 0.1%, 25.6%, 36.6%, 53.7%, and 73.0% early apoptosis, respectively. When the concentration of anthocyanidins exceeded 300 μg/mL, the proportion of early apoptotic cells decreased drastically with increasing concentration; for instance, only 2.2% of cells were early apoptotic cells at 800 μg/mL (Figure 5E1). In addition, the proportion of late apoptotic/necrotic cells increased gradually at concentrations less than 300 μg/mL and then increased rapidly, reaching 69.4% at 800 μg/mL. The proportion of total apoptotic cells increased sharply with increasing concentration, reaching a maximum level (92.4%) at 300 μg/mL, and thereafter maintained a constant level. A generally similar effect was also observed with the B16-F10 cells exposed to blueberry anthocyanins (Figure 5B1–B6 and E2). At concentrations less than 400 μg/mL, the early apoptotic cells increased rapidly with increasing concentration, and reached the maximum (74.8%) at 400 μg/mL. Thereafter, the early apoptotic cells decreased, while the late apoptotic/necrotic cells increased drastically. The total apoptotic cells increased gradually at concentrations of 0–800 μg/mL in a dose-dependent manner. Compared with the blueberry samples, doxorubicin, as the positive control, induced higher proportions of late apoptotic/necrotic cells than early apoptotic cells (Figure 5D1–D3 and E3). These results revealed that blueberry anthocyanidins and anthocyanins at suitable concentrations could effectively induce B16-F10 cell death following early and late apoptosis, rather than accidental uncontrolled cell death (necrosis).

Cell apoptosis is mediated by a variety of apoptotic proteins. Polyphenolic-induced apoptosis has been reported to be associated with the caspase family, the levels of p53, Bax, Bcl-2, Bcl-Xl [32], and so on. To further investigate the molecular mechanisms involved in the apoptosis induced by blueberry anthocyans in B16-F10 cells, two key apoptosis-related proteins (caspase-3 and p53) were analyzed using immunofluorescence methods (Figure S3). The green fluorescence intensity represents the expression level of caspase-3 and p53. The immunofluorescence assay showed that both blueberry anthocyanidin- and anthocyanin-treated groups at 200 and 400 μg/mL showed much stronger fluorescence intensity (*p* < 0.05) than the control groups (Figures S3–S6), suggesting that blueberry anthocyans had up-regulated the expression of caspase-3 and p53 in B16-F10 cells. Thus, these findings reveal that blueberry anthocyanidin and anthocyanin extracts can induce apoptosis in B16-F10 cells through up-regulation of caspase-3 and p53 levels.

## Discussion

An increasing amount of evidence from epidemiological studies has shown that diets rich in fruits or vegetables contribute to the prevention of many chronic diseases, including cancer, hyperlipidemia, cardiovascular disease, and Alzheimer’s disease [33,34]. Some researchers have recognized that diets supplemented with pure or limited compounds do not have the same health benefits as diets rich in fruits and vegetables, and the combination of phytochemicals could better exert their powerful antitumor, anticancer activities [35]. Blueberries contain a diverse range of phytochemicals (e.g. flavonols, phenolic acids, tannins, and anthocyanins) with biological functions such as anticancer, anti-inflammatory, and antioxidant activities. Among the different fractions of blueberry extract, anthocyanins were found to be the most effective inhibitors against cancerous cells [36].

Since a single anthocyanin or a few anthocyanins may lose their bioactivity or not behave in the same way as the compounds in natural fruits and vegetables, it is more valuable to investigate the anticancer activities of blueberry anthocyanin – mixtures than pure anthocyanins. In the current study, the anthocyanin extracts rich in De, Mv, and Pt glycosides were prepared from Chinese wild blueberries (with ~62% purity) by column chromatography techniques. Subsequently, the anthocyanidin extracts (with ~75% purity) containing five aglycones (De, Mv, Pt, Cy, and Pn) were obtained through acid hydrolysis and solid-phase extraction. From the MTT results, we found that blueberry anthocyanins at concentrations of 50–800 μg/mL and anthocyanidins from 50 to 400 μg/mL showed outstanding inhibitory activity against B16-F10 cells and exhibited a dose-dependent pattern. The IC_50_ values of anthocyanidins for B16-F10 cells at 24, 48, and 72 h were significantly lower (*p* < 0.05) than those of anthocyanins. It was also found that anthocyanidins showed higher inhibitory effects on HL-60 leukemia cancer cells, human hepatoma cells, and HCT-15 cells compared to anthocyanins [37]. The anticancer mechanisms of both anthocyanins and anthocyanidins can be divided into three aspects: antioxidation, molecular mechanisms related to anticarcinogenesis, and molecular mechanisms involved in proapoptosis in tumor cells [38,39]. The prominent growth-inhibitory effects of anthocyanidins on cancer cells are likely to be attributable to the free hydroxyl group at the 3-position in the flavylium moiety instead of the substitutions by various sugar moieties in the respective anthocyanins. It is worth noting that both blueberry anthocyanidins and anthocyanins at concentrations of 12.5–800 μg/mL proved to be more selective against B16-F10 cells than against normal mouse L929 fibroblast cells. In contrast, doxorubicin at concentrations of 0.04–5.0 μg/mL exhibited much stronger cytotoxicity against both B16-F10 cells and L929 cells than the two blueberry samples. However, severe side effects of doxorubicin have limited its application in high-dose chemotherapy or combined therapy with other antitumor drugs for malignant melanoma [2]. Thus, blueberry anthocyanidins and anthocyanins may attract increasing interest regarding their pharmaceutical function owing to their predominant inhibitory effects on B16-F10 cells.

The calcein-AM/PI staining results indicated that blueberry anthocyanidins and anthocyanins exerted a strong cytotoxicity on B16-F10 cells. These results also indicated that anthocyanidins showed greater inhibitory effects than anthocyanins on B16-F10 cells, which is generally consistent with the MTT results. Morphological observation showed that the increasing concentration of anthocyanidins or anthocyanins led to a clear morphological alteration of B16-F10 cells; thus, we deduced that anthocyanins and anthocyanidins could induce cell death. Since apoptosis and necrosis are two options for cell death in multicellular organisms, these results reveal that anthocyanidins and anthocyanins induced cell apoptosis or necrosis.

To further investigate the underlying inhibitory mechanism of anthocyanidins and anthocyanins on B16-F10 cells, cell cycle arrest and cell apoptosis were assessed using flow cytometry. The data showed that anthocyanidins at concentrations < 200 μg/mL induced a block at the G0/G1 phase of B16-F10 cells, while at higher concentrations, the main blockage occurred at the G2/M phase. Similarly, anthocyanins at concentrations < 400 μg/mL arrested the B16-F10 cells at the G0/G1 phase. These findings are generally consistent with the cell cycle effects of anthocyanin-rich strawberry [32] and black raspberry [40] demonstrated in other cancer cell lines, where the cell cycle blockage occurred mainly at the G0/G1 and G2/M phases. Several studies also attributed the inhibitory effects of anthocyanins on cell proliferation to their ability to block various phases of the cell cycle via effects on cell cycle regulatory proteins (e.g. p53, p21, p27, and cyclins A, B, D1, and E), and the G0/G1 phase arrest may be due to the down-regulation of cyclins D1 and E, as well as the up-regulation of p21 and p27 expression in HT-29 and COLO 320DM cells [9,31]. This study also revealed that blueberry anthocyans could block the progression of the cell cycle at the G0/G1 phase by down-regulating cyclin D1 expression in B16-F10 cells.

Apoptosis is another way in which anthocyanidins and anthocyanins can inhibit carcinogenesis. In this study, we found that anthocyanidins at concentrations of 50–300 μg/mL and anthocyanins at 50–400 μg/mL could induce early apoptosis of B16-F10 cells in a dose-dependent manner. Thereafter, at higher concentrations, the proportions of late apoptotic/necrotic cells increased sharply. This result suggests that anthocyanidins and anthocyanins could induce early apoptosis at a low or appropriate level, but promote late apoptosis or necrosis in high doses. The apoptosis-inducing effects of cyanidin-3-*O*-glucoside on two human leukemia cells could be attributed to the up-regulation of p53 and bax and down-regulation of Bcl-2 expression [41]. Mulberry anthocyanins could induce apoptosis in AGS cells through p38/p53 and p38/c-jun signaling pathways [42]. This study suggests that the proapoptotic activities of blueberry anthocyanidins and anthocyanins involve the up-regulation of caspase-3 and p53 expression. Since apoptosis is a complicated process involving many factors, the underlying mechanism involved in apoptosis induction by blueberry anthocyanidins and anthocyanins needs further investigation.

## Conclusions

Our study found that both anthocyanidin and anthocyanin extracts from blueberries could inhibit metastatic murine melanoma cell proliferation by blocking cell cycle progression and inducing apoptotic death. Anthocyanidin extracts were found to be more potent inhibitors of tumor cell proliferation than anthocyanin extracts. Moreover, the blueberry anthocyanin and anthocyanidin extracts are potential raw materials for the production of antitumor health foods and medicines. Without doubt, *in vivo* animal studies or human clinical studies are more convincing in this area. Further studies are needed to clarify the possible mechanisms and to evaluate the bioavailability of anthocyanidins in blueberries before they can be used extensively in clinical applications to reduce tumor and cancer risk.

## Supplementary Material

Supporting_information.doc
