# Supplementary material for: Antiproliferative and proapoptotic activities of anthocyanin and anthocyanidin extracts from blueberry fruits on B16-F10 melanoma cells
Source: Food Nutr Res. 2017 Jun 19;61(1):1325308. doi: 10.1080/16546628.2017.1325308 (PMC5492086; doi:10.1080/16546628.2017.1325308)
Supplement: Supporting_information.doc [file zfnr_a_1325308_sm9390.doc]

Antiproliferative and proapoptotic activities of anthocyanin and anthocyanidin extracts from blueberry fruits on B16-F10 melanoma cells

Erlei Wang^1^, Yanjun Liu^1^, Caina Xu^2^ and Jingbo Liu^1^*

^1^ College of Food Science and Engineering, Jilin University, Changchun, Jilin, China;

^2^ Key Laboratory of Polymer Ecomaterials, Changchun Institute of Applied Chemistry, Chinese Academy of Sciences, Changchun, Jilin, China

Corresponding author: Jingbo Liu; Address: College of Food Science and Engineering, Jilin University, Changchun 130062, Jilin, China; Tel: +86 431 87836351/13944170656; Fax: +86 431 87835760; E-mail: ljb168@sohu.com

Supporting method:

**(1) Immunofluorescence Staining**

To examine the expression of some of the major cell cycle regulators and apoptotic markers, The immunofluorescence staining was employed . Briefly, the B16-F10 cells were seeded on the coverslips in 6-well plates with a density of 2.0 × 10^5^ cells per well, cultured for 24 h. Then the cells were treated with various concentrations of anthocyanin and anthocyanidin extracts (0, 200, 400 μg/mL), respectively. After incubation for 24 h, the cells were washed 3 three times with PBS and fixed with 4% (w/v) paraformaldehyde for 30 min at room temperature. Cells were washed with PBS 3 times, and then permeabilized with 0.1% Triton X-100 (Sigma-Aldrich) in PBS for 5 min, washed with PBS 3 times, followed by blocking with 10% normal goat serum for 20 min. Cells were incubated with primary antibodies (p53, caspase-3 and cyclin D1) overnight at 4 °C in order to assess the pathological and immunological changes in tumor cells. The cells were then incubated with FITC (green color) goat anti-rabbit immunoglobulin G (IgG) secondary antibody (ABClonal, Boston, USA) for 40 min at 37 °C. Subsequently, the cellular nuclei were stained with 1 mL of 0.2 μg/mL of 4',6-dia- midino-2-phenylindole (DAPI, blue color) ( Life Technologies, USA) for 10 min at 37 °C. The cells were washed 3 times with PBS and the immunofluorescence staining results were observed with a confocal laser scanning microscopy (CLSM) (LSM 780, Carl Zeiss, Jena, Germany). The fluorescence intensity was quantified and analyzed by ImageJ 1.84v software (Wayne Rasband, NIH, USA).

Supporting Figures:

**Fig. S1.** Characteristic expression of cyclin D1 on B16-F10 cells induced by blueberry anthocyanidins (A) and anthocyanins (B). The fluorescence images were observed by CLSM after immunofluorescence staining. Green color indicates the expression of cyclin D1 in B16-F10 cells, and blue color indicates nuclear DAPI staining. White scale bars=20 μm.

**Fig. S2.** Fluorescence intensity ratio of cyclin D1 expression on B16-F10 cells induced by blueberry anthocyanidins and anthocyanins. The fluorescence images were observed by CLSM after immunofluorescence staining and the fluorescence intensity ratio was quantified and analyzed by ImageJ software. * indicates significant difference at p < 0.05.

**Fig. S3.** Characteristic expression of caspase-3 on B16-F10 cells induced by blueberry anthocyanidins (A) and anthocyanins (B). The fluorescence images were observed by CLSM after immunofluorescence staining. Green color indicates the expression of caspase-3 in B16-F10 cells, and blue color indicates nuclear DAPI staining. White scale bars=20 μm.

**Fig. S4.** Fluorescence intensity ratio of caspase-3 expression on B16-F10 cells induced by blueberry anthocyanidins and anthocyanins. The fluorescence images were observed by CLSM after immunofluorescence staining and the fluorescence intensity ratio was quantified and analyzed by ImageJ software. * indicates significant difference at p < 0.05.

**Fig. S5.** Characteristic expression of p53 on B16-F10 cells induced by blueberry anthocyanidins (A) and anthocyanins (B). The fluorescence images were observed by CLSM after immunofluorescence staining. Green color indicates the expression of p53 in B16-F10 cells, and blue color indicates nuclear DAPI staining. White scale bars=20 μm.

**Fig. S6.** Fluorescence intensity ratio of p53 expression on B16-F10 cells induced by blueberry anthocyanidins and anthocyanins. The fluorescence images were observed by CLSM after immunofluorescence staining and the fluorescence intensity ratio was quantified and analyzed by ImageJ software. * indicates significant difference at p < 0.05.

Supporting references:

1. Skogs M, Stadler C, Schutten R, Hjelmare M, Gnann C, Björk L, Poser I, Hyman AA, Uhlén M, Lundberg EK. Antibody validation in bioimaging applications based on endogenous expression of tagged proteins. J Proteome Res 2017; 16:147–55.

2. Lu SH, Tsai WS, Chang YH, Chou TY, Pang ST, Lin PH, Tsai CM, Chang Y C. Identifying cancer origin using circulating tumor cells. Cancer Biol Ther 2016; 17: 430–8.
